# Norovirus in Pediatric Gastroenteritis: A Study in Argentine Hospitals Before and After the Introduction of Universal Rotavirus Vaccination

**DOI:** 10.3390/vaccines13111080

**Published:** 2025-10-22

**Authors:** Karina A. Gomes, Karina A. Rivero, Christian Barrios Mathieur, Juan I. Degiuseppe, Paulo R. Cortes, Patricia A. Gonzalez, Abel Zurschmitten, María P. Castro, Viviana Parreño, Marina V. Mozgovoj, Juan A. Stupka

**Affiliations:** 1 Laboratory of Viral Gastroenteritis-National Institute for Infectious Diseases (INEI)-ANLIS “Dr. Carlos G. Malbrán” Ciudad Autónoma de Buenos Aires, Buenos Aires 1281, Argentina; krivero@anlis.gob.ar (K.A.R.); cbarrios@anlis.gob.ar (C.B.M.); jdegiuseppe@anlis.gob.ar (J.I.D.); jstupka@anlis.gob.ar (J.A.S.); 2 Microbiology Laboratory, “Del Niño Jesús” Children’s Hospital, Córdoba X5000HTT, Argentina; paulocortes19@gmail.com (P.R.C.); patogonzal895@gmail.com (P.A.G.); 3 Laboratory Section, “Junín de los Andes” Hospital, Neuquén Q8371, Argentina; abelz72@hotmail.com (A.Z.); castro.ma.pa@gmail.com (M.P.C.); 4 Institute of Virology and Technological Innovations (IVIT), Research Center for Veterinary and Agronomic Sciences (CICVyA), National Institute of Agricultural Technology (INTA), Buenos Aires C1033AAE, Argentina; vivipar3015@gmail.com; 5 Institute of Food Technology, National Institute of Agricultural Technology (INTA), Buenos Aires C1033AAE, Argentina; mozgovoj.marina@inta.gob.ar; 6 Institute of Science and Technology for Sustainable Food Systems (INCUI-NTA), Experimental Unit for Development and Demonstration (UEDD), UEDD INTA-CONICET, Buenos Aires C1033AAE, Argentina

**Keywords:** norovirus, pediatric gastroenteritis, rotavirus vaccine

## Abstract

Norovirus (NoV) is a leading cause of acute gastroenteritis (AGE) in young children worldwide. Following the introduction of universal rotavirus (RVA) vaccination in Argentina in 2015, the role of NoV in pediatric AGE warrants evaluation. This study aimed to assess the prevalence, clinical characteristics, and molecular diversity of NoV in children under five years of age, comparing the periods before and after RVA vaccine implementation. Methods: A descriptive observational study was conducted in two pediatric hospitals in Argentina. Stool samples were obtained from both outpatient and hospitalized children presenting with acute gastroenteritis (AGE) during two distinct one-year periods: 285 samples from the pre-vaccination period (2011–2012) and 212 samples from the post-vaccination period (2019–2020). NoV, RVA and other viral enteropathogens were detected by RT-qPCR or immunoassay. Positive NoV samples were genotyped by Sanger sequencing of the ORF1/ORF2 junction. Results: NoV was detected in 30.1% (86/285) and 23.5% (50/212) of cases in the pre- and post-vaccination periods, respectively. Children under two years of age and inpatients had significantly higher NoV detection in both periods. NoV mono-infections were more frequent in post-vaccination period (72% vs. 50%). NoV GII predominated in both periods, with increased genotype diversity observed post-vaccination, including GII.3[P12], GII.4 Sydney[P16], GII.6[P7], and GII.2[P16]. Conclusions: NoV remains a major cause of pediatric AGE in Argentina, particularly in children under two years old. Although NoV prevalence did not increase after RVA vaccine introduction, its clinical relevance persists. Continued molecular surveillance is essential to monitor genotype dynamics and implement prevention strategies.

## 1. Introduction

Acute gastroenteritis (AGE) in children represents a significant cause of morbidity and mortality in developing countries. According to the World Health Organization (WHO), nearly 1.7 billion cases of childhood diarrheal disease and approximately 443,832 deaths among children under 5 years of age occur worldwide each year [1].

In Argentina, the incidence of acute diarrhea cases reached approximately 1,200,000 per year, with half of these reported in children under the age of five. Additionally, during the period from 2005 to 2013, only in the public sector, there were approximately 2,000,000 hospital discharges in this age group, of which 9.2% were diagnosed with intestinal infection [2]. The significant role of viral agents in the etiology of pediatric AGE is widely recognized, especially in children younger than five years old [3]. Among these agents, *Rotavirus A* (RVA) and *Norovirus* (NoV) are reported as the most frequently detected viral agents or acute gastroenteritis followed by other viruses such as enteric *Adenovirus* (AdV), *Astrovirus* (AsV), and *Sapovirus* (SaV) [4]. NoV is considered the leading cause of AGE outbreaks across all age groups, with a particularly significant burden on young children, older adults, and immunocompromised individuals [5,6]. Clinically, NoV infection manifests as an acute, self-limiting intestinal disease characterized by the sudden onset of vomiting and diarrhea, often accompanied by abdominal cramps and fever [7]. The incubation period ranges from 12 to 72 h, and symptoms usually resolve within 1 to 3 days. However, in older adults, infants and immunocompromised individuals, gastroenteritis may be more severe and lead to complications such as dehydration, which can pose a serious threat to life [5,6,8]. NoV is a non-enveloped, icosahedral virus, with a single-stranded RNA genome of approximately 7.5 kb, organized into three open reading frames (ORFs). ORF1 encodes a polyprotein that is cleaved to yield the set of non-structural proteins, including the RNA-dependent RNA polymerase (RdRp). ORF2 encodes the major capsid protein (VP1), while ORF3 encodes for the minor capsid protein (VP2). NoV is classified into ten genogroups, of which genogroup I (GI), II (GII), IV (GIV), VIII (GVIII), and IX (GIX) can infect humans [9]. These genogroups are further subdivided into 49 genotypes and 60 P-types based on the amino acid sequence of VP1 and RdRp, respectively. Among these, GII is the most prevalent worldwide, with GII.4 being the most epidemiologically significant variant. GII.4 strains have been associated with the majority of global NoV outbreaks over the past two decades, largely due to the emergence of new variants such as Sydney 2012 and New Orleans 2009 that exhibit antigenic drift and increased transmission potential [10,11]. In Argentina, RVA has historically been the leading cause of severe diarrhea in children under five years of age. A study conducted between 1996 and 1998 in nine Argentine cities reported that RVA accounted for 42% of hospitalizations due to diarrhea in young children [12]. Following the introduction of the RVA vaccine in 2015, the incidence of severe RVA cases decreased significantly [13]. Regarding NoV, a study at the “Ricardo Gutiérrez” Children’s Hospital in Buenos Aires between 2017 and 2021, which included only outpatient children, found that 24.4% of children with acute gastroenteritis were infected with NoV [14]. The epidemiological landscape of acute gastroenteritis (AGE) has changed markedly since the introduction of national rotavirus (RVA) immunization programs worldwide in 2006 [15]. A meta-analysis demonstrated that RVA vaccination reduced the risk of severe AGE by 69% and hospitalizations by 89% [16]. Countries adopting this strategy have consistently reported sustained declines in both the incidence and severity of RVA-related AGE among children under five [17,18,19]. In Argentina, universal RVA vaccination was implemented in 2015 with the monovalent G1P [8] human vaccine (Rotarix™, GlaxoSmithKline Biologicals, Rixensart, Belgium) [20], leading to a 54% reduction in AGE cases and a 39.5% decrease in hospitalizations among children under two [13]. This major public health achievement, however, has revealed new dynamics in the prevalence of other enteric pathogens. Following RVA vaccine implementation, studies from several countries have documented changes in the leading causes of AGE. In Latin America, research in Bolivia and Nicaragua showed that norovirus (NoV) gained importance after RVA vaccine introduction, becoming the predominant cause of gastroenteritis in children under five, whereas in countries such as Finland and Canada, no changes in NoV epidemiology were observed. This differences in the epidemiological burden of NoV across countries are attributed to variations in RVA vaccine coverage, health surveillance systems, and the diversity of circulating NoV genotypes, which influence whether NoV emerges as the leading cause of pediatric AGE [21,22,23,24].Indirect mechanisms have been proposed through which RVA vaccination may influence NoV epidemiology, including reduced competition for susceptible hosts, and shifts in the balance of enteric viral pathogens [25]. Despite the growing global understanding of the role of NoV in AGE, comprehensive epidemiological studies investigating its contribution in Argentina remain scarce. Although data on NoV associated outbreaks are available in the country [26,27,28,29], there is limited information regarding its role in sporadic AGE cases among children under five years of age. The aim of this study was to evaluate the impact of NoV to sporadic AGE in children under five years of age, both hospitalized and outpatient, from two hospitals located one in the Pampa and the other in the Patagonia region, Argentina. The epidemiological shifts on NoV-associated diarrhea was evaluated by comparing the pre- and post-vaccination periods following the introduction of RVA vaccine into the national immunization program in 2015.

## 2. Materials and Methods

### 2.1. Study Design

A descriptive, observational study was conducted over two distinct periods, delineated by the implementation of the rotavirus (RVA) vaccine into the Argentinean National Immunization Program in 2015. The pre-vaccination period spanned from December 2011 to November 2012, and the post-vaccination period from March 2019 to February 2020.

### 2.2. Study Population

Children under 5 years of age with symptoms of acute gastroenteritis who were seen at outpatient clinics or hospitalized at either the ‘Del Niño Jesús’ Pediatric Hospital in Córdoba Province (a high-complexity national referral center specializing in pediatrics, located in the city of Córdoba, within the Pampa region) and the ‘Junín de los Andes’ Hospital in Neuquén Province (a medium-complexity regional center that provides care for both pediatric and adult patients, located in the Patagonian region) were included. All samples were collected as part of a The National Laboratory Surveillance Network for Viral Gastroenteritis. Case selection was based on a sentinel unit strategy, with all hospitalized cases and the first five outpatient cases per week included for each period. Exclusion criteria included the following: diarrhea lasting more than 15 days at the time of consultation; evidence of diarrhea secondary to non-infectious gastrointestinal disorders (e.g., inflammatory bowel disease, pseudomembranous colitis); immunocompromised status; age over five years; follow-up visits for relapses of previous diarrheal episodes. This study was conducted in accordance with the principles outlined in the Declaration of Helsinki. All data were anonymized. No identifiable data were collected to ensure patient confidentiality and privacy.

### 2.3. Samples

A total of 497 stool samples from children under 5 years of age were analyzed, of which 115 were collected at the “Junín de los Andes” Hospital and 382 at the “Del Niño Jesús” Pediatric Hospital in Córdoba. At Junín de los Andes Hospital, the samples included 34 from hospitalized children and 81 from outpatients. At the “Del Niño Jesús” Hospital, 152 samples were obtained from inpatients and 230 from outpatients across both study periods. During the pre-vaccine period, 98 samples were collected in Junín de los Andes Hospital and 187 in “Del Niño Jesús Hospital”. In the post-vaccine period, 17 samples were obtained in “Junin de los Andes” Hospital and 195 in “Del Niño Jesús “Pediatric Hospital. Stool samples were obtained from patients presenting with acute gastroenteritis, defined as ≥2 episodes of watery diarrhea within 24 h, with or without vomiting. In both periods, samples were collected within the first 24 h of hospitalization or within 48 h of outpatient consultation. For each case, an epidemiological data form was completed, recording: age, sex, patient status (inpatient or outpatient), date of symptoms onset date of sample collection, and RVA vaccination status. Samples were sent to the National Reference Laboratory for Rotavirus and Norovirus (LNR) at INEI-ANLIS “Dr. Carlos G. Malbrán” and stored at −80 °C until processing. A 10% suspension of fecal samples was prepared using sterile phosphate-buffered saline (PBS) or saline solution. Nucleic acids were extracted using the QIAamp Viral RNA Mini Kit, (QIAGEN Hilden, Germany) and RNA extracts were stored at −20 °C. Detection of NoV genogroups I and II was performed by RT-qPCR [30]. Detection of co-infections included sapovirus and astrovirus by multiplex RT-qPCR [10,31], enteric adenovirus by qPCR [32], and rotavirus A nanobody-based enzyme immunoassay [33]. All PCR analyses were carried out using an ABI 7500 thermocycler (Applied Biosystems, Thermo Fisher Scientific, Foster City, CA, USA). Positive NoV samples were further characterized by end-point RT-PCR and partial Sanger dideoxy sequencing of a fragment spanning the 3′ end of the RNA-dependent RNA polymerase (RdRp) and the 5′ end of the VP1 gene (ORF1/ORF2 junction) [34]. Genotypes were assigned using the Automated Norovirus Genotyping Tool [35] and further confirmed by phylogenetic analysis. The nucleotide sequences reported in this paper were submitted to the Gen Bank database under the accession numbers PV454447 to PV454475. The sequences were aligned using BioEdit version 7.2.5 and compared with prototype sequences retrieved from Gen Bank using the NCBI Blastn tool (https://blast.ncbi.nlm.nih.gov) accessed in 17 March 2025 with default parameters Blast search [36]. Phylogenetic analyses were carried out using Kimura 2 parameters model of nucleotide substitution. Phylogenetic tree was constructed by the “Neighbor-joining method” using MEGA version 11 [37]. The statistical significance of the trees was obtaining by bootstrapping values (1000 pseudo replicates). The nucleotide identity percentages were calculated based on the VP1 fragment using the MEGA version 11 software. Additionally, bacterial stool cultures were performed at each hospital using conventional culture techniques for *Salmonella* spp., *Shigella* spp., and *Campylobacter* spp., as previously described [38]. These results were recorded on the epidemiological forms and submitted to the National Reference Laboratory.

### 2.4. Statistical Analysis

To calculate association between qualitative variables, the Fisher test of proportions comparison was used, considering statistically significant a *p*-value < 0.05. Odds ratios (OR) with 95% confidence intervals were calculated to assess potentials associations between NoV detection and selected variables, such as age group (≤2 years old vs. >2 years old), sex (male vs. female), and clinical settings (inpatient vs. outpatient) [39].

## 3. Results

### 3.1. Overall NoV Detection

The overall detection of NoV was 30.1% (86/285) in the pre-vaccination period and 23.5% (50/212) in the post-vaccination period, with no significant difference between periods (OR = 1.40, 95% CI: 0.93–2.10; *p* = 0.105). The distribution of NoV infections and coinfections differed between the two study periods. NoV monoinfections were significantly more frequent in the post-vaccination period compared with the pre-vaccination period (72.0% vs. 50.0%; OR = 0.39, 95% CI: 0.18–0.82; *p* = 0.0187). In contrast, NoV/RVA coinfections decreased significantly after RVA vaccine introduction (10.0% vs. 25.6%; OR = 3.09, 95% CI: 1.09–8.78; *p* = 0.0433). Other coinfections, including NoV with enteric AdV, *Campylobacter*, *Shigella*, or *Salmonella*, were detected at low frequencies and did not show significant differences between periods. Some combinations were rare or absent, precluding statistical analysis (Table 1).

### 3.2. NoV in Outpatients and Hospitalized Patients

In the pre-vaccination period, 50% (43/86) of NoV-positive samples were detected in hospitalized patients, with 51.1% (22/43) of these representing monoinfections. Among outpatients, monoinfections were identified in 48.8% (21/43) of the samples. Notably, 93% (40/43) of NoV-positive hospitalized cases occurred in children under 2 years of age. In the post-vaccination period, 62% (31/50) of NoV-positive samples were found in hospitalized patients. Of these a high proportion, specifically 83.9% (26/31) were monoinfections. Among outpatients, monoinfections were observed in 52.6% (10/19) of the samples. As seen in the pre-vaccination period, the majority of NoV positive, 87% of hospitalized cases (37/43) occurred in children under 2 years of age. Despite the similar proportion of NoV-positive cases detected among inpatients and outpatients in the pre-vaccination period, the odds of testing positive for NoV were significantly higher in hospitalized children. This is supported by an odds ratio of 2.11 (95% CI: 1.26–3.54 *p*-value = 0.0052), indicating that children with diarrhea testing positive for NoV suffer a more severe diarrhea, that required more than twice the inpatient care than the children suffering diarrhea by other agents. In the post-vaccination period, this association became even stronger (OR: 3.85; 95% CI: 2.00–7.52; *p* < 0.001), suggesting a growing role of NoV as a cause of more severe gastroenteritis requiring hospitalization after the implementation of rotavirus vaccination (Table 2).

### 3.3. Norovirus Detection by Age Group

In the pre-vaccination period, 88.34% (76/86) of NoV-positive samples were from patients under 2 years of age. Of these, 51.3% (39/76) were monoinfections. Among patients older than 2 years, NoV monoinfection accounted for 40% (4/10) of the analyzed samples. In both age groups, the most frequent coinfection was NoV/RVA.

Notably, NoV detection was significantly more frequent in children under 2 years of age, as evidenced by an odds ratio of 2.42 (95% CI: 1.16–5.04, *p* = 0.016), indicating that children under 2 years old had more risk of NoV infection compared to older children during the pre-vaccination period (Table 2). In the post-vaccination period, 90% (45/50) of the NoV-positive samples were also detected in children under 2 years of age, and monoinfections represented a higher proportion among them (71.2% 32/45). For patients older than 2 years old, 80% (4/5) of NoV-positive cases were monoinfections. The strength of association between age under 2 years and NoV infection increased in the post-vaccination period, with an odds ratio of 4.25 (95% CI: 1.60–11.35, *p* = 0.002), suggesting that young children became even more affected by NoV in the post-vaccination period (Table 2).

### 3.4. Norovirus Detection by Sex in Pre- and Post-RV Vaccination Periods

In the pre-vaccination period, NoV was detected in 34.6% of male patients (54/156), compared to 24.8% of female patients (32/129). The odds ratio (OR) for NoV positivity in males versus females was 1.60 (95% CI: 0.93–2.75) with a *p*-value = 0.091. Likewise, during the post-vaccination period, NoV was detected in 48.0% of male patients (24/119) and in 52.0% of female patients (26/93). In this period, the odds ratio for males versus females was 0.65 (95% CI: 0.35–1.22), with a *p*-value = 0.196. The absence of statistically significance indicated that the prevalence of NoV was similar in both sexes (Table 2).

### 3.5. Distribution of NoV in Children Vaccinated and Unvaccinated Against RVA

Between December 2011 and November 2012, none of the studied cases had received any dose of the RVA vaccine. In contrast, during the post-vaccination period, vaccination status accounted for 54.7% (116/212) of the children. Among these, 82.7% (96/116) received at least one dose of the Rotarix vaccine, while 16.3% (19/116) were unvaccinated. These unvaccinated patients met the age criteria for RVA vaccination. NoV was detected in 23.9% (23/96) of vaccinated children and in 25.0% (5/20) of unvaccinated children, with no statistically significant difference observed between these groups (*p*-value = 1.00).

### 3.6. NoV Seasonality

**Figure 1:**
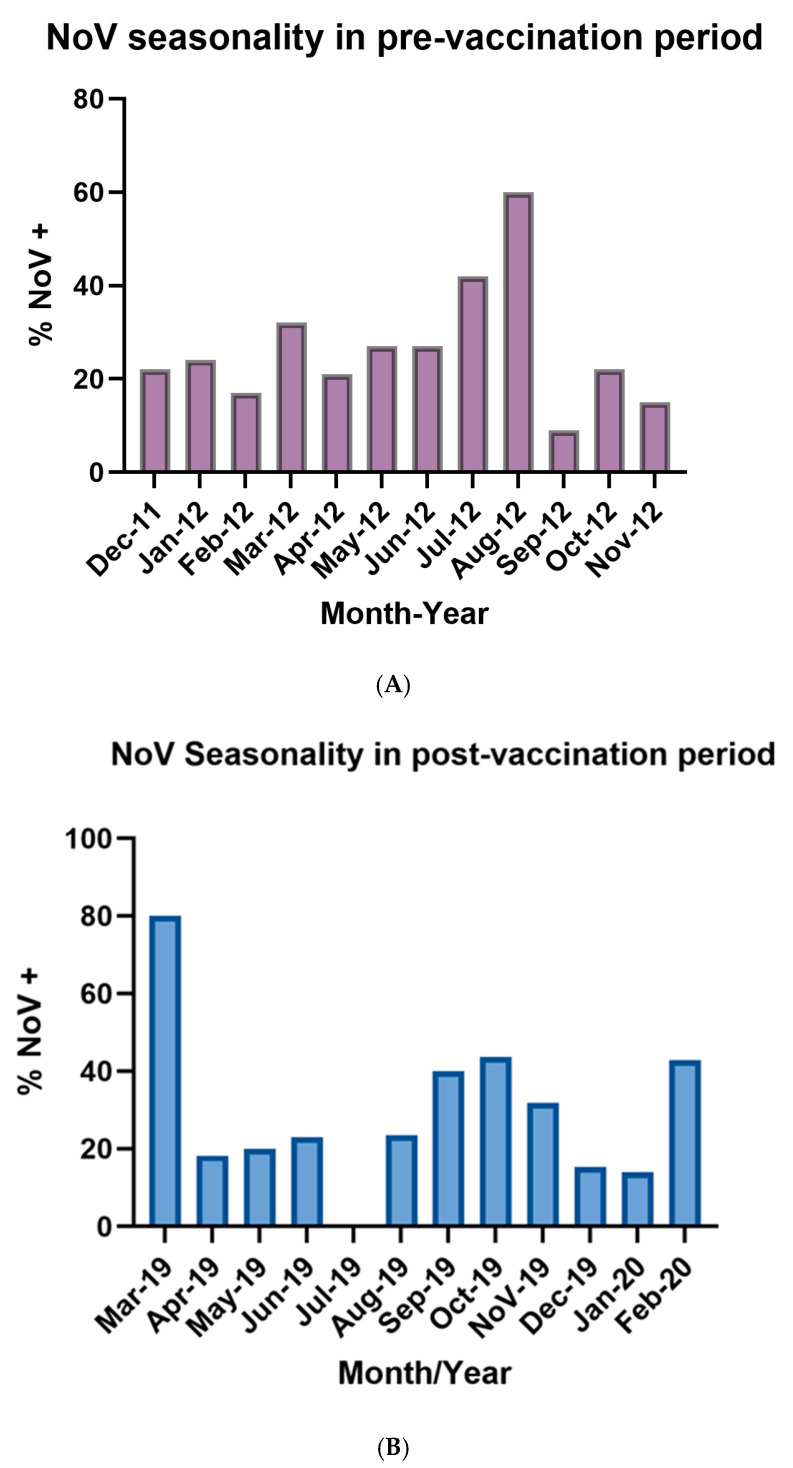
(**A**): Illustrated seasonality of NoV infections during the pre-vaccination period (December 2011–November 2012). Bars represent the monthly percentage of stool samples positive for norovirus among children under five years of age with AGE in participating centers. (**B**): Illustrated seasonality of NoV infections during the post-vaccination period (March 2019–February 2020). Bars represent the monthly percentage of stool samples positive for norovirus among children under five years of age with AGE in participating centers.

During the pre-vaccination period, the distribution of NoV-positive cases exhibited a seasonal pattern. Although cases were detected throughout the entire year, a distinct peak was observed in July and August. Overall, NoV circulation showed a seasonal pattern, with the highest activity concentrated in winter months (Figure 1A). During the post-rotavirus vaccination period, NoV positivity were registered year-round, but a pronounced peak was observed in March 2019 and a moderate peak in late winter and spring months (August to November) as well as February 2020. (Figure 1B).

### 3.7. Molecular Characterization

**Figure 2:**
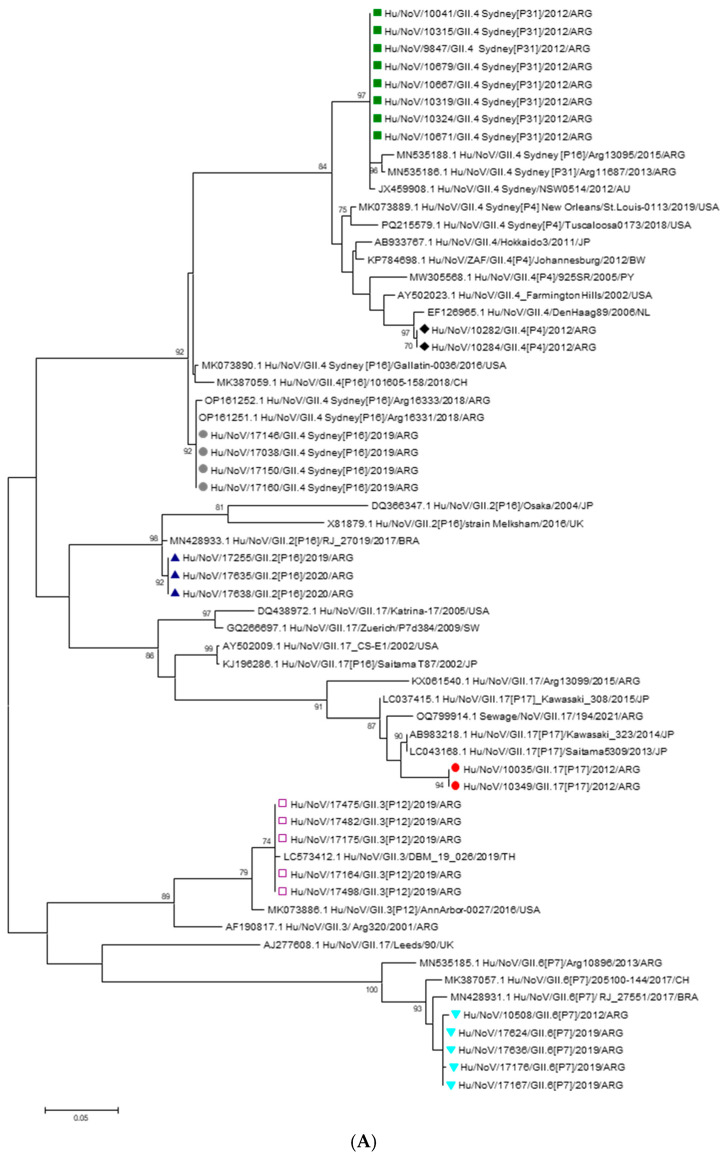

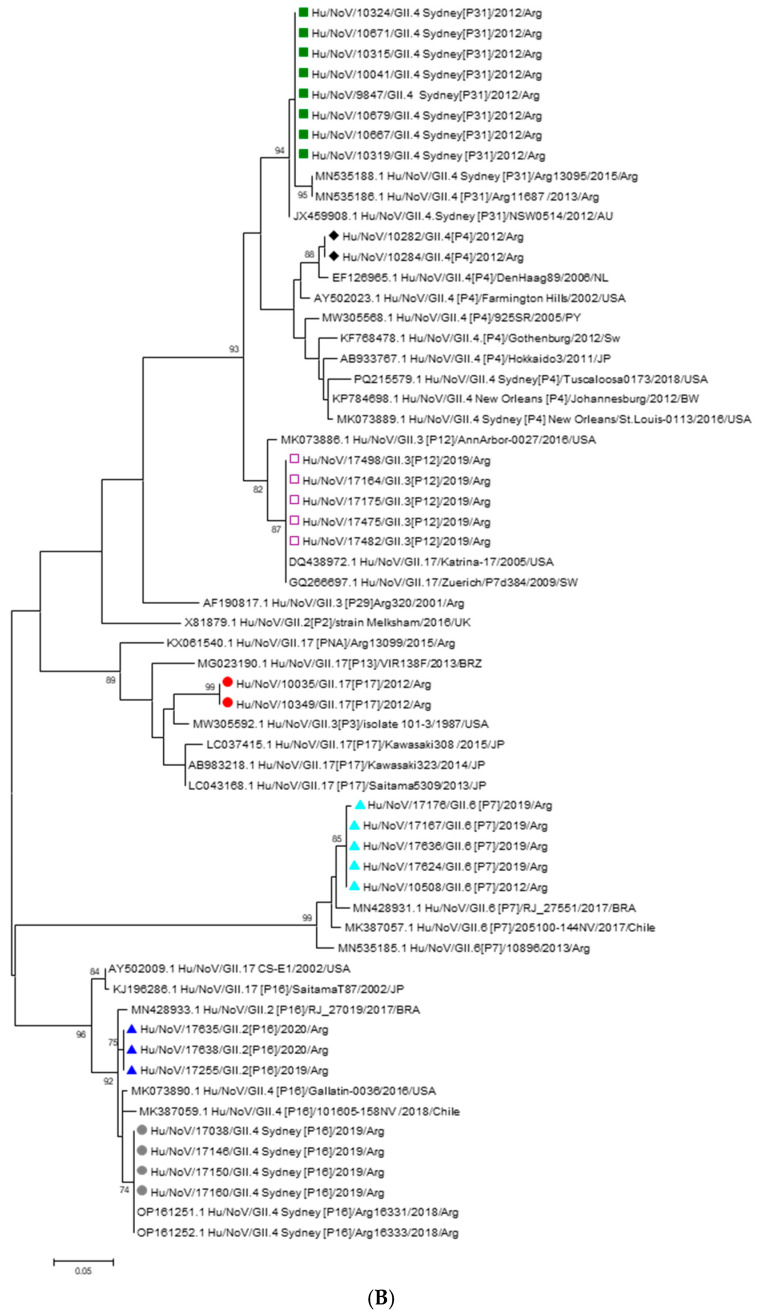
(**A**): Phylogenetic tree based on the capsid region nucleotide sequences of norovirus strains. The tree was constructed using the Maximum Likelihood method with the Kimura 2-parameter (K2P) model. Bootstrap values (1000 replicates) greater than 70% are shown at the nodes. Sequences obtained in this study are labeled as follows: 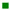 GII.4 Sydney[P31], 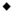 GII.4[P4], 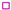 GII.3[P12], 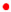 GII.17[P17], 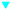 GII.6[P7], 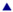 GII.2[P16], 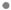 GII.4[P16]. Reference strains were retrieved from GenBank and are labeled with their accession numbers and genotypes. (**B**): Phylogenetic tree based on the RdRp region nucleotide sequences of norovirus strains. The tree was constructed using the Maximum Likelihood method with the Kimura 2-parameter (K2P) model. Bootstrap values (1000 replicates) greater than 70% are shown at the nodes. Sequences obtained in this study are labeled as follows: 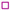 GII.3[P12], 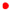 GII.17[P17], 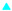 GII.6[P7], 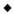 GII.4[P4], 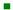 GII.4 Sydney[P31], 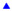 GII.2[P16], 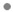 GII.4 Sydney[P16]. Reference strains were retrieved from GenBank and are labeled with their accession numbers and genotypes.

During the pre-vaccination period, genogroup identification by RT-qPCR revealed that 94.2% (81/86) of NoV-positive samples belonged to GII while 5.8% (5/86) of NoV-positive samples belonged to GI. Thirteen of these samples were successfully sequenced. Analysis of the capsid fragment revealed that 61.5% (8/13) of the sequenced samples corresponded to the NoV GII.4 Sydney 2012 variant, associated with the P31 polymerase type. Additionally, 15.4% (2/13) of the samples harbored the GII.4 Den Haag variant combined with the P4 polymerase type. The remaining characterized strains during this period were GII.6[P7] (7.7% 1/13) and GII.17[P17] (15.4%; 2/13). Most of the NoV GII.4 Sydney 2012 strains detected were from hospitalized patients. In the post-vaccination period, GII was detected in 96% (48/50), whereas 4% (2/50) corresponded to GI. Sixteen of these samples were successfully sequenced. Analysis of the capsid gene fragment identified the following variants and their respective frequencies: GII.3[P12], 31.2% (5/16); GII.4 Sydney[P16], 25% (4/16); GII.6[P7], 25% (4/16); and GII.2[P16], 18.8% (3/16). The majority of GII.4 Sydney[P16] and GII.3[P12] strains were detected in samples from hospitalized children. The analysis of the 5′ region of the VP1 gene (Figure 2A) showed that the GII.4 strains detected in 2012 and 2019, both combined with the P31 and P16 polymerases, and clustered together with the GII.4 Sydney 2012 variant strains isolated in Australia, USA and Argentina. Two GII.4 strains isolated in 2012 clustered with the Den Haag 2006 variant. The GII.2[P16] strains isolated in 2019 clustered with sequences from Japan and Brazil. The GII.3[P12] strains clustered with Argentine sequences, one of which was isolated in 2001. The GII.6[P7] strains, isolated in 2019, clustered with sequences from Brazil and Chile and with sequences isolated in pre-vaccination period. The GII.17[P17] strains detected in 2012 grouped together with the Kawasaki 323 strain, which was isolated and reported in Japan in 2013 and belongs to variant C (Figure 2A). The nucleotide identity of the strains isolated in this study compared to reference strains ranged from 87% to 99.1%, indicating that they do not represent a novel genotype or variant. Phylogenetic analysis of the 3′ region of the RNA-dependent RNA polymerase (RdRp) gene revealed that strains harboring the GII.4 Sydney capsid formed two distinct clusters, depending on their associated polymerase genotype. RdRp sequences of the P16 genotype were identified in strains with both GII.2 and GII.4 capsids. These strains clustered together with others previously reported in Argentina and neighboring Latin American countries, consistent with the regional circulation of the P16 polymerase. In contrast, strains carrying the P31 polymerase clustered with local GII.4 Sydney[P31] strains and those detected in Australia, forming a separate lineage from the GII.4 Sydney[P16] strains due to nucleotide divergence in the RdRp gene (Figure 2B).

## 4. Discussion

This is the first study to compare the role of NoV in both hospitalized and outpatient children under five years of age before and after the introduction of RVA vaccination in Argentina. All data were collected in the pre-COVID 19 pandemic period and were therefore not influenced by the SARS-CoV-2 circulation. The objective of this study was to evaluate the contribution of NoV to the etiology of gastrointestinal infections in children under five years of age across two distinct one-year periods: prior to and following the introduction of the RVA vaccine into the Argentinean national immunization program. Evidence from multiple countries in Latin America has documented a high prevalence of NoV-associated gastroenteritis in children under five years of age during the post-rotavirus vaccine period, leading to the hypothesis that NoV might have assumed the role previously dominated by RVA [40]. In our study, global NoV detection was high during both the pre- and post-vaccination periods, indicating that NoV has consistently played a major role in pediatric gastroenteritis, rather than emerging as a consequence of RVA vaccine implementation. However, the distribution of infection types shifted substantially. The higher proportion of NoV monoinfections in the post-vaccination period, together with the significant reduction in NoV/RVA coinfections, suggests that RVA vaccination may have altered the landscape of viral gastroenteritis, reducing opportunities for dual infections. This pattern aligns with the expected decline in RVA circulation following widespread vaccination and underscores the relative role of NoV as an independent pathogen in the post-vaccine era. The increase in NoV relevance in the post-vaccine era may rather reflect the reduced contribution of RVA to the overall burden of viral gastroenteritis, making the relative importance of NoV more evident. Our findings are consistent with observations from other settings, such as Finland, where no significant change in global NoV prevalence was reported after rotavirus vaccine introduction [23], and Canada, which also reported high NoV frequency in both periods [24]. Furthermore, the higher detection of NoV observed in the pre-vaccination period in our study may be partially explained by the emergence and global spread of the GII.4 Sydney 2012 variant [41]. This variant was first identified in Australia in early 2012 and rapidly became predominant worldwide, replacing previously circulating strains such as GII.4 New Orleans 2009 [42]. Its emergence coincided with a significant increase in the number of outbreaks and sporadic cases of gastroenteritis across multiple continents, likely due to antigenic changes that enabled immune escape and enhanced transmissibility [41,43]. The dominance of GII.4 Sydney 2012 contributed to a notable rise in norovirus-related disease burden globally during that year [44]. In both periods, NoV detection was significantly higher in children under 2 years of age. The risk of NoV infection was higher in children aged 6 months to 2 years in both periods. These findings suggest that NoV continues affect children under 2 years of age, regardless the vaccination for RVA [23,45]. These findings are consistent with reports from other countries, such as Ghana, and agree with previous data reported in our country [14,46]. The elevated frequency of NoV in this age group may be attributed to several factors, including hand-to-mouth behavior, inadequate hygiene practices, close contact in daycare settings, and an immature immune system. NoV showed a strong association with hospitalization in both analyzed periods and hospital settings. The odds of hospitalization were significantly higher among NoV-positive patients, especially in the post vaccination period although no significant differences were observed between both periods. In Argentina, there are no direct comparative studies evaluating NoV severity or hospitalization rates before and after the introduction of the RVA vaccine. A study conducted in a hospital in Buenos Aires highlighted the importance of NoV in children under five years of age, but it was performed during the post-vaccine period and included only outpatient cases [14]. In agreement with our results a population-based study from Israel, reported no significant increase in NoV-associated hospitalizations following RVA vaccine introduction [47]. Similarly, a Latin American meta-analysis of pediatric hospitalizations found comparable NoV prevalence before and after RVA vaccination [40]. These findings support our results which evidence that NoV remains a major cause of severe disease in children, regardless of changes in RVA epidemiology which has changed due to RVA vaccine implementation [23,48].

Coinfections involving viral and bacterial enteropathogens are frequently observed in pediatric gastroenteritis [49]. In many instances, these coinfections are associated with the consumption of unsafe or contaminated water or food. Unsafe water and food not only facilitates the simultaneous transmission of multiple pathogens but also reflects broader deficiencies in sanitation and access to clean water, which remain critical determinants of enteric disease burden in vulnerable populations [50]. In this study the most common combination in both periods was NoV and RVA. However, the incidence of this coinfection decreased significantly in the post-vaccination period, likely attributable to the introduction of the RVA vaccine. Regarding NoV coinfections with bacterial pathogens, the most frequently detected was with *Campylobacter* spp. This coinfection has been reported as common in children; however, it remains unclear which of the two pathogens played the primary causative role or acted as a contributing factor to the infection [51,52].

Interestingly, no significant difference in NoV detection was observed between RVA-vaccinated and unvaccinated children under 5 years old during the post-vaccine period.

This finding is critical as it highlights the independent epidemiology of NoV, suggesting that the implementation of RVA vaccination does not substantially alter NoV circulation or individual susceptibility to infection. Unlike some reports that describe shifts in the enteric viral landscape post-RVA vaccine introduction [15,45], our data indicate that the benefits of RVA vaccine remain specific to preventing RVA disease without indirectly impacting the burden of NoV. Furthermore, our results do not support the idea of AGE caused by RVA to be supplanted by NoV after vaccine introduction, at least in these two hospitals under study with different demographic properties. It should also be considered that the relative increase in NoV detection post-vaccination may reflect an artifact due to the decline in RVA cases, resulting in NoV representing a larger share of AGE cases rather than a true rise in incidence.

Regarding seasonal pattern of NoV infections during the pre-vaccination period, NoV infections exhibited peaks, with the highest proportion of positive cases occurring in the winter months of July and August and early spring, consistent with the well-established seasonality of NoV in temperate regions of the southern hemisphere [53]. It should be clarified that in Argentina, the winter season extends from June to August, spring from September to November, summer from December to February, and autumn from March to May. This winter predominance is likely influenced by factors such as increased viral stability in cooler temperatures and behavioral patterns that facilitate transmission, including more indoor crowding [54]. Whether or climate change could influence the seasonal patterns of NoV infection by impacting its transmission, geographic distribution, and prevalence, has not yet been considered [54]. In the post vaccination period, a very high positivity rate was observed in March 2019; however, this finding should be interpreted with caution since it was based on a small number of samples in that month and may not reflect a true epidemiological peak. Although NoV cases were detected more evenly throughout the year, a moderate peak was observed from August to November, coinciding with late winter and spring. This change could be due to modifications in environmental factors such as changes in temperature and humidity, which may influence the stability and transmission of the virus [55]. During 2011–2012 and 2019–2020, meteorological records in Córdoba and Neuquén reported seasonal fluctuations in temperature and humidity consistent with regional norms. Moreover, the “El Niño” phenomenon led to increased precipitation during spring, which may have altered relative humidity and local temperatures, thereby creating environmental conditions more favorable for NoV persistence and transmission [56]. Additionally, the circulation of distinct NoV genotypes or variants may contribute to shifts in NoV seasonality, as different genotypes exhibit unique seasonal peaks influenced by environmental and climatic factors [53,57]. Although there are no conclusive studies linking RVA vaccination directly with seasonal changes in NoV circulation, some studies show that after the introduction of the RVA vaccine, the relative proportion of cases attributed to NoV in children increases [48]. Molecular characterization revealed the predominance of GII, consistent with global data identifying GII as the main cause of human gastroenteritis [55]. During the pre-vaccination period, GII.4 Sydney[P31] was dominant, particularly among hospitalized children under 2 years old, consistent with its recognized association with severe disease and global prevalence since 2012 [44]. GII.4 Den Haag[P4] and GII.17[P17] were also detected, with the latter clustering with the Kawasaki 2013 strain, reflecting its international spread [58,59]. Post-vaccination, greater diversity was observed, with GII.3[P12], GII.4 Sydney[P16], and GII.6[P7] circulating at similar frequencies, alongside GII.2[P16]. The detection of GII.4 Sydney with both P31 and P16 genotypes highlights ongoing recombination, a key mechanism driving its adaptability [60]. Phylogenetic analyses showed similarity with globally circulating strains, suggesting both international introduction and local persistence, as also reported for GII.6[P7] and GII.3[P12] [61,62,63]. The high similarity of the Argentine strains to reference sequences suggests that no novel genotypes or variants emerged during the study period. This observation is consistent with previous reports indicating the global circulation of these established genotypes, highlighting that the diversity observed in Argentina reflects the broader known variability rather than local emergence of new variants [62]. The diminished competition from a dominant pathogen like RVA could potentially create a more permissive environment, allowing the diversification and increased detection of less common NoV genotypes or novel recombinants that might otherwise have been less frequently observed [63]. Detection of several genotypes among children under five years old further emphasizes the clinical significance of these strains and underscores the need for ongoing molecular surveillance to detect emerging variants with epidemic potential, especially considering that young children may serve as reservoirs for the emergence and dissemination of novel variants. This study confirms the continued dominance of NoV GII in pediatric gastroenteritis and reveals dynamic shifts in circulating NoV genotypes over time. The emergence and persistence of recombinant GII.4 Sydney variants, along with increased genetic diversity in the post-vaccination period, highlight the evolving epidemiology of NoV in the context of universal RVA vaccination. This study has several limitations. Only a small number of NoV-positive samples could be successfully sequenced, possibly due to repeated freeze–thaw cycles that may have compromised nucleic acid integrity. Additionally, the RT-PCR assay used to amplify the target region may not have had sufficient sensitivity, which could have led to an underestimation of the true genetic diversity of circulating strains or the failure to detect less common variants. This study was limited to two sentinel hospitals, which may not fully reflect the national epidemiology of NoV. The results from the post-vaccination period were obtained prior to the COVID-19 pandemic. Since then, the distribution of enteric viruses may have changed.

## 5. Conclusions

NoV remains a leading cause of pediatric gastroenteritis in Argentina, affecting primarily children under two years of age, regardless of RVA vaccine implementation. Although RVA vaccination reduced NoV/RVA coinfections, it did not change overall NoV prevalence or severity. NoV GII continued to dominate, with dynamic genotype shifts and recombinant variants observed post-vaccination. Changes in NoV circulation may be multifactorial, including variations in circulating genotypes, climatic influences, and the indirect impact of RVA vaccination. These findings highlight the independent epidemiology of NoV and the need for continued molecular surveillance to detect emerging strains and inform public health strategies.

## Figures and Tables

**Table 1:** Comparison of Norovirus Monoinfections and Coinfections in Pre- and Post-RVA Vaccination Periods.

Type of NoV Infection	Pre-Vaccination Period	Post-Vaccination Period	OR (95% CI)	*p*-Value
NoV monoinfection	43/86 (50.0%)	36/50 (72.0%)	0.39 (0.18–0.82)	0.0187
NoV/RVA	22/86 (25.6%)	5/50 (10.0%)	3.09 (1.09–8.78)	0.0433
NoV/AdV	8/86 (9.3%)	2/50 (4.0%)	2.46 (0.50–12.08)	0.3239
NoV/AdV/SV	1/86 (1.2%)	0/50 (0.0%)	NA	NA
NoV/AdV/RVA	2/86 (2.3%)	0/50 (0.0%)	NA	NA
NoV/*Campylobacter*	7/86 (8.1%)	3/50 (6.0%)	1.38 (0.33–4.95)	0.7453
NoV/*Shigella*	2/86 (2.3%)	2/50 (4.0%)	0.22 (0.04–1.14)	0.1054
NoV/*Salmonella*	1/86 (1.2%)	0/50 (0.0%)	NA	NA
NoV/RVA/AdV/*Campylobacter*	0/86 (0.0%)	1/50 (2.0%)	NA	NA
NoV/AdV/*Shigella*	0/86 (0.0%)	1/50 (2.0%)	NA	NA

OR: Odds ratio, CI: Confidence interval, NA: not applicable.

**Table 2:** Comparison of NoV detection by population characteristics: Pre- and Post-RVA vaccination periods.

Variable	Category	Period	NoV Positive n (%)	NoV Negative n (%)	OR (95%CI)	*p*-Value
Care setting	Inpatients	Pre	43 (50.0%)	64 (32.1%)	2.11 (1.26–3.54)	0.005
	Outpatients	Pre	43 (50.0%)	135(67.8%)	—	—
	Inpatients	Post	31 (62.0%)	48 (29.6%)	3.85 (2.00–7.52)	<0.001
	Outpatients	Post	19 (38.0%)	114 (70.4%)	—	—
Age group	<2 years	Pre	76 (88.3%)	151 (75.8%)	2.42 (1.16–5.04)	0.016
	≥2 years	Pre	10 (11.6%)	48 (24.1%)	—	—
	<2 years	Post	45 (90.0%)	110 (67.9%)	4.25 (1.60–11.35)	0.002
	≥2 years	Post	5 (10.0%)	52 (32.1%)	—	—
Sex	Male	Pre	54 (34.6%)	102 (65.4%)	1.60 (0.93–2.75)	0.091
	Female	Pre	32 (24.8%)	97 (75.2%)	—	—
	Male	Post	24 (48.0%)	95 (58.6%)	0.65 (0.35–1.22)	0.196
	Female	Post	26 (52.0%)	67 (41.4%)	—	—

OR: Odds ratio; CI: Confidence interval. Comparisons were made between paired categories (Inpatients vs. Outpatients, <2 years vs. ≥2 years, Male vs. Female). OR and *p*-values are indicated for each group.

## Data Availability

The GenBank accession numbers for the sequences obtained for this study are PV454447-PV454475.

## Abbreviations

The following abbreviations are used in this manuscript:

NoV	Norovirus
SaV	Sapovirus
RVA	Rotavirus
AdV	Adenovirus
AsV	Astrovirus
ORF	Open reading frames
RdRp	RNA-dependent RNA polymerase
